# A Water-Soluble Polysaccharide from the Fibrous Root of *Anemarrhena asphodeloides* Bge. and Its Immune Enhancement Effect in Vivo and in Vitro

**DOI:** 10.1155/2022/8723119

**Published:** 2022-09-10

**Authors:** Qi Huang, Jingwen Wang, Rumin Zong, Deling Wu, Chuanshan Jin

**Affiliations:** ^1^Department of Pharmacy, Anhui University of Chinese Medicine, Hefei 230012, China; ^2^Anhui Province Key Laboratory of Traditional Chinese Medicine Decoction Pieces of New Manufacturing Technology, Hefei 230012, China; ^3^Traditional Chinese Medicine Processing and Inheritance Base of National Administration of Traditional Chinese Medicine (Anhui University of Chinese Medicine), Hefei 230012, China; ^4^Anhui Province Key Laboratory of Pharmaceutical Preparation Technology and Application, Hefei 230012, China

## Abstract

**Background:**

The fibrous roots of *Anemarrhena asphodeloides* Bge. (FRAAB) are byproducts of the rhizome of *Anemarrhena asphodeloides*. Some studies have revealed secondary metabolic small molecules in FRAAB, but there are few reports on the polysaccharides of FRAAB (PFRAAB). *Aim of the Study.* The present study aimed to investigate the preliminary characterization and underlying mechanism of immune stimulation of PFRAAB.

**Materials and Methods:**

The crude polysaccharide of FRAAB was obtained by hot water extraction and alcohol precipitation, and PFRAAB was purified by a diethylaminoethyl-52 (DEAE-52) cellulose chromatographic column and graphene dialysis membrane. The preliminary characterization of PFRAAB was studied by ultraviolet (UV) scanning and Fourier Transform Infrared Reflection (FTIR). The molecular weight and composition of PFRAAB were analysed by high-performance gel permeation chromatography (HPGPC) and high-performance liquid chromatography (HPLC), respectively. The immune stimulation of PFRAAB was investigated by using cyclophosphamide- (CCP-) treated mice and RAW264.7 cells.

**Results:**

A water-soluble PFRAAB was obtained with a molecular weight of 115 kDa and was mainly composed of arabinose (ara), galactose (gal), glucose (glc), and mannose (man). Compared with CCP-induced mice, PFRAAB significantly (*p* < 0.05 or *p* < 0.01) increased the spleen and thymus index, ameliorated injury to the spleen and thymus, and evaluated immunoglobulin levels. In addition, PFRAAB also increased the secretion of nitric oxide (NO), interleukin-1*β* (IL-1*β*), tumour necrosis factor-*α* (TNF-*α*), and IL-6 in RAW264.7 cells and upregulated the expression of toll-like receptor 4 (TLR4), Myd88, nuclear factor kappa-B (NF-*κ*B) P65, p–NF–*κ*B P65, IKB-*α*, and p-IKB-*α*.

**Conclusion:**

PFRAAB possesses immune stimulation activity and can be used as a potential resource for immune-enhancing drugs. Our present study provides a scientific basis for the comprehensive development of *Anemarrhena asphodeloides* medicinal plant resources.

## 1. Introduction

The immune system plays an important role in human health and is considered a protective barrier against ageing or apoptotic cells in the body, foreign pathogenic microorganisms, and other external factors [1]. Immunosuppression refers to immune dysfunction, which is commonly caused by bacterial or viral infection, chemical drugs, or negative emotions. Diseases such as upper respiratory tract infection, intestinal infection, or even tumour could be caused by immunosuppression [2, 3]. In immunosuppressive organisms, the growth of immune organs, including the thymus and spleen, is inhibited, and the levels of natural killer (NK) cells and immunoglobulin in the serum decrease [4]. In addition, the level of CD3/CD4 lymphocytes also decreased due to immunosuppression [5].

RAW264.7 cells, one of the macrophages, play an important role in the innate immune system. RAW264.7 cells can directly engulf pathogens or indirectly secrete inflammatory factors such as TNF-*α* and interleukin-6 (IL-6) to improve the body's immunity [6]. RAW264.7 cells are the ideal in vitro model and can be used to investigate the mechanism of immune enhancement [7].

Currently, levamisole and glucans have been used as immunomodulators in treating immunosuppression in the clinic, but there are still some side effects, such as neutropenia and fever [8]. Fortunately, polysaccharides, one of the most important plant secondary metabolites, have attracted a great deal of scientific interest for their good merits of immune enhancement and few side effects [9].

The dried rhizome of *Anemarrhena asphodeloide*s Bge. (AAB), which belongs to the Liliaceae family, is a traditional Chinese medicine (TCM) with antipyretic, hypoglycaemic, and immunoregulatory effects [10–12]. The dried rhizome of AAB was also approved as food by the National Health Commission of People's Republic of China in 2018. AAB is mainly distributed in Hebei, Anhui, and Shanxi, and the total output of the rhizome of AAB was approximately 50,000 tons per year. In the clinical practice of TCM, only the rhizome of AAB is used. The fibrous roots of dried rhizome of AAB (FRAAB) are the byproducts in the processing of dried rhizome of AAB and account for 1/3 of the total output of dried rhizome of AAB. However, the FRAAB were usually discarded during the primary processing. Studies have shown that there are still some effective active ingredients in FRAAB [13]. If the FRAAB could be used reasonably, it would provide a new drug source and reduce the waste of medicinal plant resources.

In the present study, a water-soluble polysaccharide from FRAAB (PFRAAB) was extracted and purified, and the preliminary characterization and monosaccharide composition of PFRAAB were studied. The immune enhancement effect of PFRAAB was studied on cyclophosphamide- (CCP-) induced immunosuppressive mice. The pathological changes in the spleen and thymus, the level of WBCs, NK-cell activity, immunoglobulin, and CD3/CD4 T lymphocytes in different experimental groups were used to estimate the in vivo immune enhancement of PFRAAB. Furthermore, RAW264.7 cells were used to investigate the underlying mechanism of immune enhancement of PFRAAB related to TLR4-NF-*κ*B.

To the best of our knowledge, this is the first study to reveal the properties and immune enhancement effect of PFRAAB.

## 2. Materials and Methods

### 2.1. Materials and Chemicals

The FRAAB were collected from Bozhou City (Anhui Province, China) in October 2021 and were identified by Professor Chuanshan Jin. The voucher specimen is kept in the Laboratory of Natural Pharmaceutical Chemistry (Anhui University of Chinese Medicine, Hefei, Anhui, China). The FRAAB samples were dried at 50°C until the water content was less than 10%, and then the samples were smashed into powder (∼80 mesh).

Cyclophosphamide (CCP, no. 2020018) was purchased from Shengdi Pharmaceutical Co., Ltd. (Jiangsu, China). Thymosin (TM, no. CC2020018) was supplied by Changchun Fuchun Pharmaceutical Co., Ltd. (Changchun, China). Monosaccharide standards, including fucose, rhamnose, arabinose, galactose, glucose, xylose, mannose, fructose, ribose, galacturonic acid, glucuronic acid, mannuronic acid, and guluronic acid, and lipopolysaccharide (LPS) were purchased from Sigma–Aldrich (St. Louis, USA). DMEM and FBS were purchased from Sparkjade Biotechnology Co., Ltd. (Shandong, China) and Gibco (Grand Island, USA), respectively. ELISA kits for NO, TNF-*α*, IL-1*β*, and IL-6 were purchased from Beyotime (Shanghai, China), and RAW264.7 cells were purchased from Saibai Kang Biotechnology Co., Ltd. (Shanghai, China).

Antibodies against TLR4, Myd88, NF-*κ*B p65, p–NF–*κ*B p65, IKB-*α*, and p-IKB-*α* were purchased from Abcam (Beijing, China); TAK-242 (TLR4 inhibitor) was purchased from Apexbio (Houston, USA). The distilled water was filtered with a Milli-Q water purification system (Milford, MA, USA). The Sevage reagent was composed of chloroform and *n*-butanol with a volume ratio of 1 : 4, and chloroform and *n*-butanol were purchased from Tianjin Siyou Company (Tianjin, China).

### 2.2. Extraction and Purification of PFRAAB

The powder of FRAAB was accurately weighed, and distilled water was added to the FRAAB powder at a ratio of 20 : 1 (liquid–solid). The mixture was refluxed in a heat water bath 3 times for 1 h each time. After refluxing, the extraction was collected and concentrated to 1/10 of the original volume, and the concentrated extraction was precipitated with ethanol at a final concentration of 80% (v/v) and then stored at 2°C for 12 h to obtain the biomacromolecules. Subsequently, the precipitates were treated with sevage reagent to remove the protein, and crude polysaccharides (CP) were obtained. The CP solution was dialyzed by a graphene dialysis membrane with a retained molecular weight of 3.5 kDa. Finally, the refined PFRAAB was prepared by freeze drying for further experiments. The ratio of the yield from the FRAAB powder to the refined PFRAAB was 2.31%.

### 2.3. Preliminary Characterization of PFRAAB

#### 2.3.1. UV Scanning of PFRAAB

For UV spectrum analysis, a total of 2.12 mg PFRAAB was weighed precisely and dissolved in 25 mL distilled water, and the PFRAAB solution (0.085 mg mL^−1^) was scanned by a UV-8000 ultraviolet spectrophotometer (Yu'an Analysis Instrument Co., Ltd., Shanghai, China) in the wavelength range of 200∼400 nm.

#### 2.3.2. Fourier Transform Infrared Spectroscopy (FTIR) Detection

The IR spectrogram of PFRAAB was obtained by the potassium bromide (KBr) method according to Alliouche's methods with modification [14]. Briefly, a total of 100 mg dry KBr was added to the PFRAAB powder (2.0 mg), and then the mixture was pressed and screened by a Nicolet 6700 Fourier Transform Infrared Reflection (FTIR) spectrometer (Thermo Fisher Scientific, USA) at 4000–500 cm^−1^ at a resolution of 2 cm^−1^. Another 100 mg KBr was used to exclude background interference.

#### 2.3.3. Determination of Molecular Weight

The molecular weight of PFRAAB was determined by HPGPC equipment (ICS5000 Ion Chromatography System, Thermo Fisher Scientific, USA) combined with a refractive index detector (RI-10A, Thermo Fisher Scientific, USA). The chromatographic analysis was performed on a BRT105-104-102 tandem gel column (8 × 300 mm), and the NaCl solution (0.05 M) was selected as the mobile phase. The flow rate was 0.6 mL min^−1,^ and the column temperature was 40°C. Dextran standards with molecular weights of 5.0 kDa (no. 20200561), 11.6 kDa (no. 20200563), 23.8 kDa (no. 20200564), 48.6 kDa (no. 20200565), 80.9 kDa (no. 20200566), 148 kDa (no. 20200568), 273 kDa (no. 20200569), 409 kDa (no. 20200570), and 667 kDa (no. 20200571) were used to establish the standard curve, which were all supplied by Sigma Company (USA).

A total of 5.05 mg PFRAAB was weighed precisely and dissolved in 1 mL distilled water to prepare the PFRAAB solution. The PFRAAB solution was filtered with a 0.45 *µ*m membrane prior to HPGPC analysis, and the injection volume was 20 *µ*L. The molecular weight of PFRAAB was calculated according to the standard curve.

#### 2.3.4. Monosaccharide Composition Analysis

A total of 2 M trifluoroacetic acid solution (TFA) was added to the PFRAAB sample (5 ± 0.05 mg) and hydrolysed at 120°C for 2 hours. Then, methanol was added to the mixture, and the acid was removed by evaporation 3 times. The hydrolysed sample was dissolved in sterile water, and the solution was filtered with a 0.22 *µ*m membrane for HPLC analysis. The monosaccharide composition analysis was conducted on high-performance liquid chromatography (HPLC) combined with refractive index (RI) detection and a DionexTM CarboPacTM PA20 (150 × 3.0 mm, 10 *μ*m) column. The elution gradient of mobile phase A (0.1 M NaOH) and mobile phase B (0.1 M NaOH, 0.2 M NaAc) was as follows: 0 min A/B (95 : 5 V/V), 30 min A/B (80 : 20 V/V), 30.1 min A/B (60 : 40 V/V), 45 min A/B phase (60 : 40 V/V), 45.1 min A/B phase (95 : 5 V/V), and 60 min A/B phase (95 : 5 V/V). The flow rate and column temperature were 0.5 mL/min and 40°C, respectively.

### 2.4. FRAAB Exerted Immune Enhancement Activity in Vivo

#### 2.4.1. Animals and Experimental Groups

A total of 60 healthy Kunming mice (18 ± 2 g, Laboratory Animal Certificate no. SCXK2021-128) were purchased from the Experimental Animal Center of Anhui Medical University (Anhui, China). All of the mice were kept under a temperature of 23–25°C and humidity of 50–60%. The animal house was in a 12/12 h dark and light cycle (lights on 6:30 am). The mice had free access to standard laboratory food and water. All of the mice were reared adaptively for 4 days before the establishment of the immunosuppressive model.

The immunosuppressive animal model was established by intraperitoneal injection with CCP (100 mg kg^−1^) once a day for 3 consecutive days. Thymosin (TM) was selected as the positive control drug at a dose of 10 mg kg^−1^. The mice injected with CCP were randomly divided into 5 groups: the model group (CCP group), positive drug (TM, 10 mg kg^−1^) group, PFRAAB low-dose (PFRBBA-L, 125 mg kg^−1^) group, PFRAAB medium-dose (PFRBBA-M, 250 mg kg^−1^) group, and PFRAAB high-dose (PFRBBA-H, 500 mg kg^−1^) group.

There were 10 mice in each group. Another 10 healthy mice were selected as the normal control (NC) group. All mice in the PFRAAB groups and positive drug group were given corresponding doses of PFRAAB or TM at 9:00 am and 17:00, and each mouse in the normal control group was administered the same volume of physiological saline. The administration lasted for 14 days.

All animal experiments were approved by the animal ethics committee of Anhui University of Traditional Medicine (Animal Ethics no. AHTCM-2021125).

#### 2.4.2. Determination of Peripheral White Blood Cell (WBC) Count, NK-Cell Activity, and Immunoglobulins in Serum

On the 14th day of PFRAAB administration, peripheral blood was collected from the eyes of each mouse, and the white blood cells were counted by an automatic blood cell analysis instrument (Mindry, BC-5180 CRP, Wuhan, Hubei, China). The determination of natural killer cell activity was conducted by Han's method with some modification [15]. YAC-1 cells and splenic lymphocytes were selected as the target cells and effector cells, respectively. YAC-1 cells were seeded in a 96-well plate at a density of 2 × 10^5^ cells per well, and then splenic lymphocyte cells were added to the corresponding well at a density of 1 × 10^7^ cells per well. The 96-well plates were incubated at 37°C with 5% CO_2_ for 20 h.

The orbital blood of each mouse was collected and kept at 25°C for 2 h. Then, the blood was centrifuged at 3500 r•min^−1^ for 10 min, and the serum was obtained. The levels of IgA, IgM, and IgG were measured under the guidance of the manufacturer's instructions.

#### 2.4.3. The Spleen and Thymus Index and HE Staining

The mice were sacrificed by cervical dislocation, and then the weights of the spleen and thymus of each mouse were measured. The spleen or thymus index was the ratio of spleen or thymus weight (mg) to body weight (*g*). Pathological research was carried out by haematoxylin and eosin (HE) staining. In short, the spleen and thymus tissues were fixed in 10% (v/v) neutral buffered formaldehyde for 1 week. Then, the spleen and thymus samples were washed with PBS (1%) and cut into 5 *μ*m slices with a freezing microtome. The slices were stained with HE and scanned by fluorescence microscopy (Nikon, Shanghai, China).

#### 2.4.4. Measurement of T Lymphocyte Subsets

The splenic lymphocytes of the mice were isolated from the spleen of each mouse. Briefly, the spleen was gently ground with lymphocyte separation medium, and then the mixture was filtered with a nylon net (∼200 mesh). The filtrate was transferred to a microcentrifuge tube with 500 *μ*L RPMI-1640 medium and centrifuged at 4°C and 2000 r min^−1^ for 30 min. The upper layer of the suspension was washed with 10 mL RPMI-1640 medium and centrifuged at 4°C and 1000 r·min^−1^ for 10 min. The supernatant was discarded, and the sublayer was resuspended in RPMI-1640 medium.

The concentration of splenic lymphocytes was adjusted to 1 × 10^5^ per millilitre, a total of 1 mL of cell suspension was added to a microcentrifuge tube, and 5 *μ*L of anti-CD3/CD4 was added to the tube. Each tube was shaken and incubated at 37°C with 95% O_2_ and CO_2_ for 10 min. Finally, the cells were washed twice with 500 *μ*L PBS and analysed by flow cytometry (Navios 3 L 10C, Beckman, USA), and the data were analysed with FlowJo v. 09.

### 2.5. FRAAB Exerted Immune Enhancement Activity in Vitro

#### 2.5.1. Cell Viability

RAW264.7 cells were cultured in DMEM containing 10% (v/v) FBS and 1% (v/v) penicillin–streptomycin and incubated at 37°C with 5% CO_2_ for 24 h. Cell viability was assayed by CCK-8 assay. Briefly, the cells were cultured in 96-well plates (5 × 10^5^ cells per well) for 24 h and then treated with a series concentration of PFRAAB (12.5 to 400 *μ*g•mL^−1^) and incubated for 24 h. Finally, CCK-8 solution (10 *μ*L) was added to each well, and the optical density (OD) was measured at 540 nm. The cells without PFRAAB were selected as the blank control group. The ratio of the OD value of the sample group to the OD value of the blank group was calculated as the cell viability.

#### 2.5.2. Phagocytosis Assay

The phagocytosis assay was carried out according to Pan's methods with some modifications [16]. The cell density was adjusted to 5 × 10^5^ cells per well and treated with 50, 100, and 200 *μ*g•mL^−1^ PFRAAB or LPS (1 *μ*g•mL^−1^) for 24 h. After 24 h, the supernatant was discarded, and neutral red stain (100 *μ*L) was added to each well. After coincubation for 1 h, the cells were washed with PBS 3 times, and 100 *μ*L of cell lysate was added. The absorbance was measured at 540 nm with a microplate reader. The phagocytosis rate was calculated according to the following formula.

Phagocytosis index (PI) = A1/A0 (A0: the absorbance of the blank control, A1: the absorbance of the sample).

#### 2.5.3. ELISA Analysis of IL-6, TNF-*α*, IL-1*β*, and NO

RAW264.7 cells in logarithmic growth phase were incubated with PFRAAB (50, 100, and 200 *μ*g•mL^−1^), LPS (1 *μ*g•mL^−1^), and TAK-242 for 24 h. The levels of cytokines, including IL-1*β*, IL-6, TNF-*α*, and NO, were determined by ELISA kits according to the manufacturer's instructions.

Polymyxin B (PMB), an LPS activity inhibitor, was used to determine whether there was LPS in PFRAAB [17]. RAW264.7 cells were pretreated with or without PMB (100 *μ*g•mL^−1^) for 1 h. Then, PFRAAB or LPS was used to stimulate RAW264.7 cells to secrete NO. The level of NO was determined by ELISA kits as above.

#### 2.5.4. RNA Extraction and RT–PCR Analysis

The mRNA expression of iNOS, TNF-*α*, IL-1*β*, and IL-6 was evaluated by real-time reverse transcription-polymerase chain reaction (RT–PCR). Total RNA was isolated from RAW264.7 cells by TRIzol® reagent (Sparkjade Biotechnology Co., Ltd., Shandong, China), and the RNA was reverse transcribed into cDNA by a SPARKscript II RT Plus kit (with cDNA Eraser). Then, the levels of IL-1*β*, IL-6, iNOS, and TNF-*α* expression were amplified by 2 × SYBR Green qPCR Mix (with ROX) according to the manufacturer's instructions. GAPDH was used as the internal reference, and the relative expression was calculated by the 2^−△△Ct^ method. The primers and amplification sequences are listed in Table 1.

#### 2.5.5. Western Blotting

Protein expression was detected by western blotting. After being treated with different concentrations of PFRAAB (50, 100, and 200 *μ*g•mL^−1^) and LPS (1.0 *μ*g•mL^−1^) for 24 h, the RAW264.7 cells were lysed in RIPA to extract proteins. The same amount of protein was subjected to SDS–PAGE to transfer to a PVDF membrane. Then, the membranes were incubated with specific antibodies against TLR4, Myo88, NF-*κ*B P65, p–NF–*κ*B P65, IKB-*α*, and p-IKB-*α* at 4°C overnight, and the secondary antibody was applied for 1 h at 37°C.

### 2.6. Statistics

The data of the experiments are presented as the mean ± SD. One-way ANOVA was used to compare multiple groups, and statistical analysis was performed with SPSS 21.0 statistical software (IBM, New York, NY). Differences between the two groups were compared using Dunnett's tests and were considered significant when *p* < 0.05.

## 3. Results

### 3.1. The Preliminary Characterization of PFRAAB

#### 3.1.1. UV and FTIR Scanning Results

**Figure 1:**
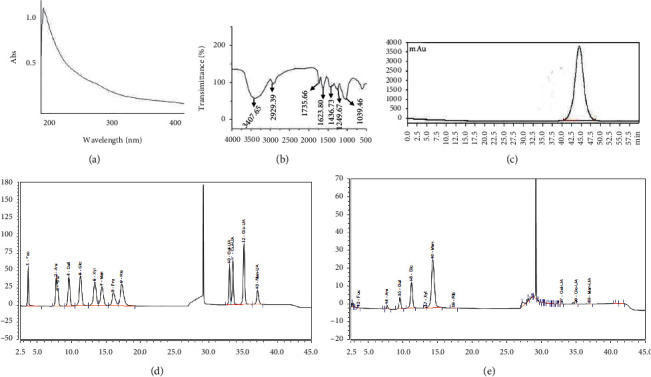
Preliminary characterization of PFRAAB. UV scanning of PFRAAB (a). FTIR scanning of PFRAAB (b). HPGPC analysis of PFRAAB (c). HPLC chromatogram of standard monosaccharides (d). HPLC chromatogram of hydrolysed PFRAAB (e).

The UV spectrum showed a smooth curve in the range of 200–400 nm except for a weak absorption at approximately 250 nm (Figure 1(a).

According to the FTIR results, the peaks at 3407.65 cm^−1^ and 2929.39 cm^−1^ were characteristic of the stretching vibrations of -OH and C–H, respectively. The wave number at 1735 cm^−1^ was the -C=*O* stretching vibration of -COOH. Furthermore, 1623.80 cm^−1^ represented the -C=C- asymmetric vibration, and 1436.73 cm^−1^ represented the C-O- stretching vibration. In addition, 1249.67 cm^−1^ and 1039.46 cm^−1^ were attributed to the angular vibration of -OH and the contractive vibration of C-O, respectively.

#### 3.1.2. The Molecular Weight of PFRAAB

The linear equation was *Y* = −0.1917*x *+* *12.108 (*R*^2^ = 0.9934), which was generated according to dextran standards with series molecular weights, as mentioned in Section 2.3.4. The molecular weight of PFRAAB was 115 kDa.

#### 3.1.3. Analysis of Monosaccharide Components of PFRAAB

After HPLC analysis, 4 main peaks were detected in PFRAAB, including arabinose (ara), galactose (gal), glucose (glc), and mannose (man), with a molar ratio of 1 : 3:6.37 : 27.2. Uronic acids, including galacturonic acid, glucuronic acid, and mannuronic acid, were also detected in PFRAAB.

### 3.2. Immune Enhancement of PFRAAB in Vivo

#### 3.2.1. PFRAAB Alleviated the Damage to the Spleen and Thymus in CCP-Induced Immunosuppressed Mice

**Figure 2:**
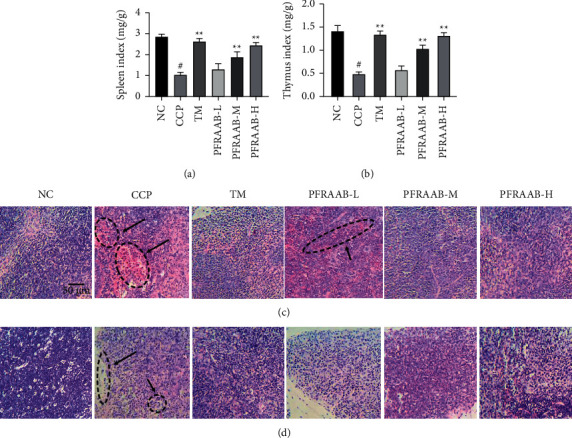
Effects of PFRAAB on the spleen or thymus index. The results of spleen index (a). Results of thymus index (b). HE staining of the spleen (c). HE staining of the thymus (d). The data are presented as the mean ± SD (*n* = 10), ^#^*p* < 0.05 compared with NC group and ^*∗∗*^*p* < 0.01 compared with CCP group. The scale bar was 50 *μ*m with magnification ×200.

As shown in Figures 2(a) and 2(b), compared with the normal control (NC) group, the spleen and thymus index decreased significantly in the CCP group (*p* < 0.05). After treatment with PRFAAB, the organ index increased significantly compared with that in the CCP group, especially in the PFRAAB-M and PFRAAB-H groups.

HE staining is shown in Figures 2(c) and 2(d). HE staining of the spleen showed that the boundary of the white and red pulp lymphoid was clear, and the spleen cells were arranged tightly and regularly with clear nuclei in the normal group. In the CCP group, the boundary of the white and red pulp lymphoid was destroyed, and part of the splenic corpuscle was sparse, while this phenomenon was reversed in the PFRAAB-M and PFRAAB-H groups (Figure 2(c). In the histopathological images of thymus HE staining (Figure 2(d), normal cortical morphology and flails can be seen in the normal control group. In the CCP group, the boundary between the medulla and cortex was not clear, and the number of medullae significantly decreased. PFRAAB remarkably alleviated thymus damage.

#### 3.2.2. PFRAAB Increased the Number of Leukocytes in Peripheral Blood, NK-Cell Activity, and Immunoglobulin in CCP-Induced Immunosuppressed Mice

**Figure 3:**
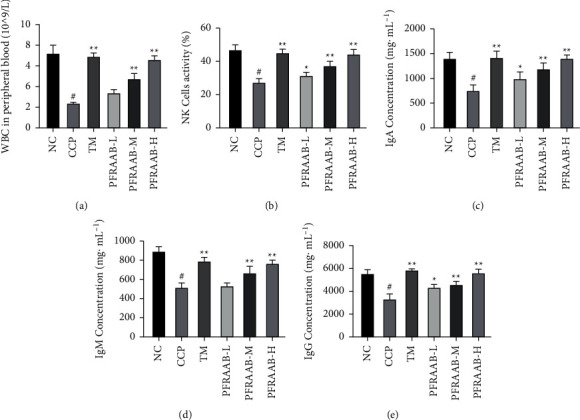
PFRAAB increased the number of WBCs, NK-cell activity, and immunoglobulin in CCP-induced immunosuppressed mice. Effect of PFRAAB on the number of WBCs (a). Effect of PFRAAB on NK-cell activity (b). Effect of PFRAAB on IgA levels (c). Effect of PFRAAB on IgM levels (d). Effect of PFRAAB on IgG levels (e). NC: normal control. The data are presented as the mean ± SD (*n* = 10), ^#^*p* < 0.05 compared with the NC group, ^*∗*^*p* < 0.05, ^*∗∗*^*p* < 0.01 compared with the CCP group.

According to Figure 3(a), the number of WBCs in the peripheral blood of the CCP group mice was 2.37 ± 0.72 (×10^9^/L) and showed a significant decrease compared with the normal control (NC) group. After treatment with PFRAAB, the number of WBCs increased significantly in the PFRAAB-M (*p* < 0.01) and PFRAAB-H (*p* < 0.01) groups compared with the CCP group. In addition, NK-cell activity also increased significantly in the PFRAAB-M (*p* < 0.01) and PFRAAB-H (*p* < 0.01) groups compared with that in the CCP group (Figure 3(b)). Moreover, compared with the normal control (NC) group, the levels of IgA, IgM, and IgG in the serum of the CCP group significantly decreased (*p* < 0.01). After treatment with PFRAAB, the level of immunoglobulin showed a significant increase in the PFRAAB group, especially at doses of 250 and 500 *μ*g•mL^−1^ (*p* < 0.01 or *p* < 0.01).

#### 3.2.3. PFRAAB Increased the Levels of CD3^+^ and CD4^+^ in CCP-Induced Immunosuppressed Mice

**Figure 4:**
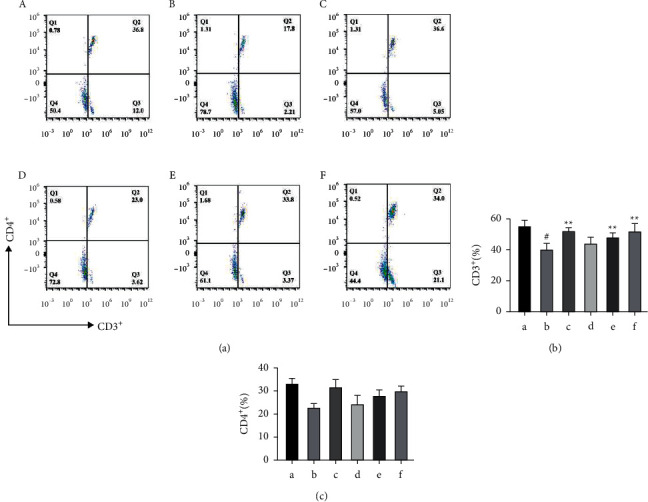
PFRAAB increased the levels of CD3^+^ and CD4^+^ in CCP-induced immunosuppressed mice. The results of flow cytometry (a). The level of CD3^+^ in peripheral blood (b). The level of CD4+ in peripheral blood (c). (a) Normal control (NC) group, (b) CCP group, (c) TM group, (d) PFRAAB-L group, (e) PFRAAB-M group, and (f) PFRAAB-H group. The data are represented as the mean ± SD (*n* = 10), ^#^*p* < 0.05 compared with the blank control group, ^*∗∗*^*p* < 0.01 compared with the CCP group.

As shown in Figure 4, compared with the CCP group, the levels of CD3^+^ and CD4^+^ significantly (*p* < 0.05) decreased. After intervention with the positive control drug and PFRAAB (especially in the medium- and high-dose groups), the levels of CD3^+^ and CD4^+^ cells increased significantly (*p* < 0.01).

### 3.3. Immune Enhancement of PFRAAB in Vitro

#### 3.3.1. Effects of PFRAAB on Cell Viability and Phagocytosis in RAW264.7 Cells

**Figure 5:**
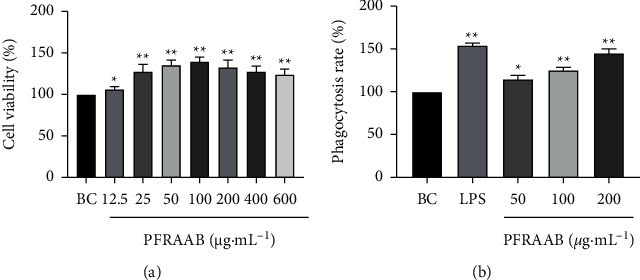
The results of the cell viability and phagocytosis assays. PFRAAB increased the cell viability (a). PFRAAB increased the phagocytosis of RAW264.7 cells (b). BC: blank control. The data are presented as the mean ± SD (*n* = 3), ^*∗*^*p* < 0.05, ^*∗∗*^*p* < 0.01 compared with the BC group.

The CCK-8 assay was used to estimate whether PFRAAB showed cytotoxic effects on RAW264.7 cells. According to Figure 5(a), all concentrations of PFRAAB in the experiment improved the cell viability when compared with the blank control (BC) group. In addition, the cell viability increased significantly (*p* < 0.05 or *p* < 0.01) when the concentration of PFRAAB was 12.5 to 100 *μ*g•mL^−1,^ and the cell viability was the highest when the concentration of PFRAAB was 100 *μ*g•mL^−1^. Unfortunately, the cell viability began to decrease when the concentration of PFRAAB reached 200 *μ*g•mL^−1^. Therefore, three concentrations (50, 100, 200 *μ*g•mL^−1^) of PFRAAB were chosen for further study.

As shown in Figure 5(b), compared with the blank control (BC) group, phagocytosis was significantly (*p* < 0.05 or *p* < 0.01) increased by the three concentrations (50, 100, 200 *μ*g•mL^−1^) of PFRAAB, and the concentration of 100 *μ*g•mL^−1^ showed the best effect.

#### 3.3.2. Effects of PFRAAB on NO and Cytokines Produced by RAW264.7 Cells

**Figure 6:**
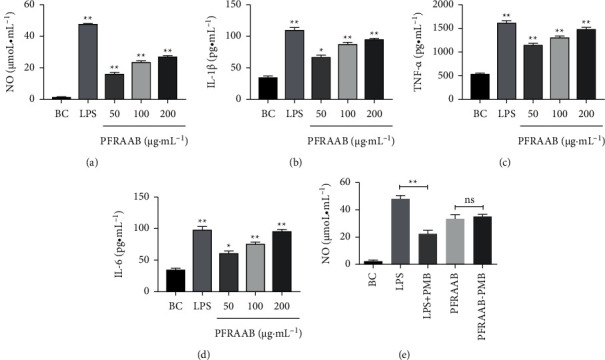
Effect of PFRAAB on the levels of NO and cytokines. Effect of PFRAAB on NO (a), IL-1*β* (b), TNF-*α* (c), and IL-6 (d), and the results of whether LPS is contained in PFRAAB (e). BC: blank control. The data are presented as the mean ± SD (*n* = 3), ^*∗*^*p* < 0.05, ^*∗∗*^*p* < 0.01 compared with the BC group.

According to Figures 6(a)–6(d), the levels of NO, TNF-*α*, IL-1*β*, and IL-6 increased significantly (*p* < 0.05 or *p* < 0.01) in the PFRAAB (50, 100, 200 *μ*g•mL^−1^) and LPS groups compared with the blank control group and showed a dose-effect relationship within a certain range. As shown in Figure 6(e), the level of NO decreased significantly in the LPS + PMB group compared with the LPS group, and there was no significant difference in the level of NO whether PMB was added to PFRAAB.

#### 3.3.3. Effect of PFRAAB on the mRNA Expression of iNOS and Cytokines in RAW264.7 Cells

**Figure 7:**
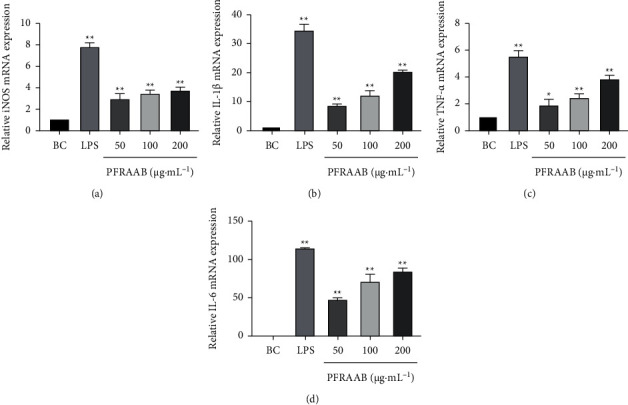
Effect of PRAAB treatment on RAW264.7 cell mRNA expression levels of iNOS (a), IL-1*β* (b), TNF-*α* (c), and IL-6 (d). Data are expressed as the means ± SD (*n* = 3). Significant differences in the PFRAAB group compared with the blank control (BC) group are shown as ^*∗*^*p* < 0.05, ^*∗∗*^*p* < 0.01.

According to Figure 7, compared with the blank control (BC) group, PFRAAB remarkably promoted the mRNA expression levels of iNOS and cytokines in RAW264.7 cells.

#### 3.3.4. PFRAAB Exerted Immune Enhancement Related to the TLR4-NF-Κb Signalling Pathway

**Figure 8:**
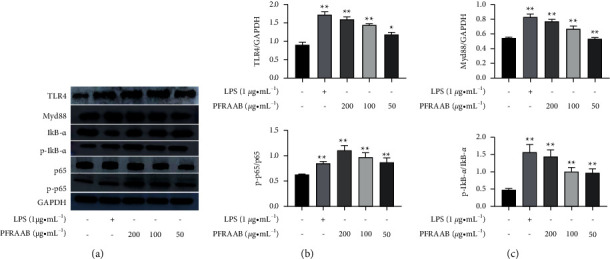
Effects of PFRAAB on TLR4 signalling pathway protein expression in RAW264.7 cells. The data are presented as the mean ± SD (^*∗*^*p* < 0.05, ^*∗∗*^*p* < 0.01 compared with the blank control group).

As shown in Figure 8, the expression of TLR4, Myd88, p-IkB-*α*/IkB-*α*, and p-p65/*p*65 was significantly (^*∗*^*p* < 0.05 or ^*∗∗*^*p* < 0.01) upregulated by three concentrations (50, 100, 200 *μ*g•mL^−1^) of PFRAAB in a dose-dependent manner. The expression of proteins related to the TLR4 signalling pathway reached the highest when the concentration of PFRAAB was 200 *μ*g•mL^−1^. Therefore, 200 *μ*g•mL^−1^ PFRAAB was selected for further experiments.

**Figure 9:**
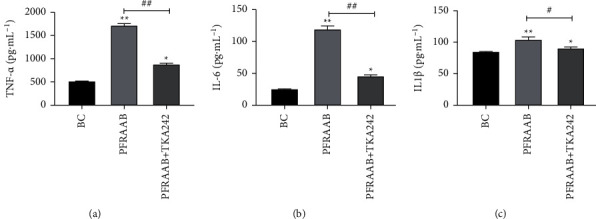
Effects of PFRAAB on the secretion of TNF-*α* (a), IL-6 (b), and IL-1*β* (c) in RAW264.7 cells via TLR4. ^*∗*^*p* < 0.05 and ^*∗∗*^*p* < 0.01 when comparing with the BC group. ^#^*p* < 0.05, ^##^*p* < 0.01 when PFRAAB group compared with PFRAAB + TKA242 group.

TAK-242 was used as an inhibitor of TLR4 to suppress the TLR4-NF-*κ*B pathway. The results showed that the secretion of TNF-*α*, IL-6, and IL-1*β* in RAW264.7 cells was markedly decreased after treatment with TAK-242 (Figure 9).

## 4. Discussion

The dried rhizome of *Anemarrhena asphodeloides* is a commonly used medicine in China with antipyretic, antitumour, and immune enhancement effects [18, 19]. FRAAB are the byproducts of the dried rhizome of *Anemarrhena asphodeloides* when processed during primary processing. Currently, a large number of FRAAB have been abandoned with the development of the TCM industry and have not attracted enough attention, which would cause a certain degree of environmental pollution and waste of resources [20]. Therefore, studying the chemical substances and bioactivities of FRAAB is of great significance to improve the utilization of medicinal resources of *Anemarrhena asphodeloides*.

Previous studies showed that the polysaccharides obtained from the dried rhizome of *Anemarrhena asphodeloides* possessed antioxidant activities, in vitro hypoglycaemic activity, and laxative effects [12, 21, 22]. In addition, the polysaccharides of AAB exert neuroprotective and immunoregulatory effects, but the molecular mechanism is unclear [10]. Moreover, literature studies have shown that some secondary metabolic small molecules, such as neomangiferin, mangiferin, isomangiferin, timosaponin BII, and timosaponin AIII, all exist in FRAAB and AAB [23], which indicates that macromolecular substances, such as polysaccharides, also exist in FRAAB. However, PFRAAB and their activity have not been reported. In this research, the preliminary chemical properties and immune enhancement of PFRAAB were investigated, which provided a scientific basis for further study of the fibrous roots of *Anemarrhena asphodeloides*. Our results suggest that PFRAAB can be used as a natural source of immune stimulation agents and provide a scientific basis for the comprehensive utilization of AAB resources.

Polysaccharides, mainly derived from natural products or TCMs, are polymers composed of several monosaccharides and linked by glycosidic bonds [24]. Polysaccharides possess high polarity and can be extracted with hot water. Meanwhile, the proteins could also be extracted by hot water, which can be removed by alcohol precipitation and membrane separation techniques [25]. In this paper, PFRAAB was extracted and purified from FRAAB. The UV scanning results showed that there was no absorption in the range of 200–400 nm except for the weak absorption at approximately 250 nm, which suggested that protein or nucleic acids were reduced to the lowest and that there were only some uronic acids in PFRAAB. Moreover, HPGPC analysis showed that there was a single and symmetric peak, which indicated that PFRAAB was a homogeneous polysaccharide. The results of HPLC analysis showed that there were uronic acids in PFRAAB, which was in accordance with the results of UV scanning.

Immune organs (spleen and thymus), immune cells, and immune proteins constitute the immune system, which is an important barrier to protect the body from the interference of external pathogenic organisms [26]. CCP, a cytotoxic immune suppressive agent, is commonly used as an antitumour agent in the clinic [27]. However, excessive CCP use could lead to the suppression or injury of immune organs, the inhibition of NK-cell activity, and a decrease in the levels of T lymphocytes and immunoglobulin [28]. In this study, the immune organ index and the levels of immune cells and immunoglobulins in CCP-induced immunosuppressed mice were all markedly decreased after treatment with CCP, but this phenomenon could be reversed by PFRAAB at doses of 250 or 500 *μ*g•mL^−1^. Our study successfully confirmed that PFRAAB exerted immune enhancement by elevating immune organs, immune cells, and immunoglobulins.

TM, a commonly used immune enhancer, was selected as the positive control. Our results revealed that there was no significant difference between the PFRAAB-H group and the TM group in elevating the number of peripheral blood WBCs, NK-cell activity, and immunoglobulin (IgA, IgG, IgM) levels induced by CCP, although the effect in the PFRAAB-H group was slightly lower than that in the TM group. In the clinic, most immune enhancers, such as TM, interferon, and nucleic acids, need to be administered by intramuscular injection to exert their efficacy [29]. The administration mode of PFRAAB was oral administration, and TM and PFRAAB were administered in different ways, which may have different requirements for the drug dose. Moreover, intramuscular injection may bring more pain to patients than oral administration. Thus, our study proved that PFRAAB was a potential oral immune enhancer. Furthermore, the literature reported that FRAAB contains small molecules with antitumour and neuroprotective effects [30] but the effects of macromolecular substances such as polysaccharides in PFRAAB have rarely been reported. Our research provides a further understanding of FRAAB.

T lymphocytes play an important role in immune response and immune recognition. CD3^+^ and CD4^+^ are helper T cells and the two main T lymphocyte subsets according to their surface markers [31]. Besides, flow cytometry is an established technology used for the determination of different types of cells [32]. The higher the level of CD3^+^ and CD4^+^, the stronger the immune function. The present study showed PFRAAB-M and PFRAAB-H group significantly increased the level of CD3^+^ and CD4^+^, which suggested that PFRAAB exerted good immune enhancement effective.

LPS, a glycolipid present on the surface of Gram-negative bacteria, is recognized as an inflammatory agent [33]. The proinflammatory factors secreted by RAW264.7 cells, such as IL-1*β*, IL-6, and TNF-*α*, increased significantly when stimulated by LPS [34]. However, cytokines, including IL-1*β*, IL-6, and TNF-*α*, play an important role in mediating and regulating the immune response, and host immunity requires these cytokines when encountering foreign harmful microorganism invasion [35]. The increasing level of IL-1*β* contributes to the promotion of the proliferation of T lymphocytes and further participates in cellular immunity [36]. IL-6 and TNF-*α* are mainly produced by macrophages and have immune activation effects to further remove infectious agents [37, 38]. In addition, the secretion of cytokines increased when given a low concentration of NO [39]. Therefore, moderately increasing the secretion of cytokines is helpful to improve immune function. In the present study, our results showed that the levels of IL-1*β*, IL-6, and TNF-*α* secreted by RAW264.7 cells increased significantly after stimulation with PFRAAB but were lower than those of the LPS group, which suggested that PFRAAB has the effect of immune stimulation and improves immune function to a certain extent. Furthermore, the mRNA expression levels of iNOS, IL-1*β*, TNF-*α*, and IL-6 in RAW264.7 cells were also elevated by PFRAAB. Moreover, PMB addition did not affect the secretion of NO by RAW264.7 cells, which indicated that there was no LPS in PFRAAB. Our results validated that PFRAAB could exert enhanced stimulation by increasing the levels of NO and cytokines.

The toll-like receptor 4 (TLR4) is one of the most important proteins in the immune system and functions in identifying pathogens [40]. Once TLR4 is activated, its downstream proteins, such as myeloid differentiation factor 88 (Myd88), are recruited, and I*κ*B-*α* is phosphorylated and hydrolysed. Finally, the NF-*κ*B pathway is activated [41], and the production of cytokines such as TNF-*α*, IL-6, and IL-1*β* is promoted by NF-*κ*B-p65 [42]. In this study, the expression of TLR4, Myd88, IkB-*α*, p-IkB-*α*, p65, and p-p65 was obviously upregulated by PFRAAB. Furthermore, TAK-242 was selected as the inhibitor of TLR4 to investigate the signalling pathway of PFRAAB. The results showed that the levels of TNF-*α*, IL-6, and IL-1*β* in RAW264.7 cells were reduced when they were treated with TAK-242, which suggested that the immune enhancement of PFRAAB was related to the TLR4-Myd88-NF-*κ*B signalling pathway.

## 5. Conclusion

In summary, a water-soluble polysaccharide was obtained from FRAAB. The preliminary chemical properties and monosaccharide composition were analysed. Subsequently, the immune enhancement of PFRAAB in vivo and in vitro was investigated. The in vivo results showed that PFRAAB could ameliorate the injury to the spleen and thymus of immunosuppressive mice induced by CCP and improve the activity of NK cells and the level of immunoglobulin. Moreover, the in vitro results showed that PFRAAB could improve the phagocytosis of RAW264.7 cells, and with no cytotoxicity, PFRAAB could also stimulate RAW264.7 cells to secrete NO and cytokines. The underlying mechanism of PFRAAB immune-enhancing activity may be related to the TLR4-NF-*κ*B signalling pathway. In summary, the results suggested that PFRAAB showed good immune enhancement and that FRAAB could be used as a source of immune enhancers. This study also supplies a scientific reference for the comprehensive utilization of *Anemarrhena asphodeloides* Bge.

## Figures and Tables

**Table 1:** Forward and reverse primers.

Gene	Length (bp)	Forward primer	Reverse primer
GAPDH	169	GCAGTGGCAAAGTGGAGATTG	CGCTCCTGGAAGATGGTGAT
iNOS	171	GGGAGAAAGCGCAAAACATTTC	ATACTGTGGACGGGTCGATG
IL-1*β*	98	GAAGAAGAGCCCATCCTCTG	TCATCTCGGAGCCTGTAGTG
IL-6	101	TGAGATCTACTCGGCAAACC	TCAGATACCTGACAACAGGA
TNF-*α*	96	CTCATGCACCACCATCAAGG	ACCTGACCACTCTCCCTTTG

## Data Availability

Data are available upon request to the authors.

## Abbreviations

AAB: *Anemarrhena asphodeloides* Bge.
CCP: Cyclophosphamide
DMEM: Dulbecco's modified Eagle medium
ELISA: Enzyme-linked immunosorbent assay
FBS: Fatal bovine serum
FRAAB: Fibrous roots of *Anemarrhena asphodeloides* Bge.
FTIR: Fourier Transform Infrared Reflection
HPGPC: High-performance gel permeation chromatography
HPLC: High-performance liquid chromatography
IL: Interleukin
LPS: Lipopolysaccharide
NF: Nuclear factor
NO: Nitric oxide
NK: Natural killer
PFRAAB: Polysaccharide of *Anemarrhena asphodeloides* Bge.
TCM: Traditional Chinese medicine
TLR4: Toll-like receptor 4
TM: Thymosin
TNF-*α*: Tumour necrosis factor-*α*
UV: Ultraviolet
WBC: White blood cell.
